# Chemical Composition of *Pinus roxburghii* Bark Volatile Oil and Validation of Its Anti-Inflammatory Activity Using Molecular Modelling and Bleomycin-Induced Inflammation in *Albino* Mice

**DOI:** 10.3390/molecules22091384

**Published:** 2017-08-29

**Authors:** Rola M. Labib, Fadia S. Youssef, Mohamed L. Ashour, Mohamed M. Abdel-Daim, Samir A. Ross

**Affiliations:** 1 Department of Pharmacognosy, Faculty of Pharmacy, Ain-Shams University, Cairo 11566, Egypt; fadiayoussef@pharma.asu.edu.eg (F.S.Y.); ashour@pharma.asu.edu.eg (M.L.A.); 2 National Center for Natural Products Research, University of Mississippi, University, MS 38677, USA; sross@olemiss.edu; 3 Department of Pharmacology, Faculty of Veterinary Medicine, Suez Canal University, Ismailia 41522, Egypt; abdeldaim.m@vet.suez.edu.eg; 4 Department of BioMolecular Sciences, School of Pharmacy, University of Mississippi, University, MS 38677, USA

**Keywords:** anti-inflammatory activity, bleomycin, gas-chromatography, molecular modelling, *Pinus roxburghii*, Pinaceae discipline

## Abstract

The chemical composition of *Pinus roxburghii* bark essential oil (PRO) was qualitatively and quantitatively determined using GC/FID and GC/MS. The anti-inflammatory activity was assessed in vitro by evaluating the binding percentages on the cannabinoids and opioids receptors. Bleomycin (BLM)-induced pulmonary inflammation in *albino* mice was adopted to assess PRO anti-inflammatory efficacy in vivo. In silico molecular modelling of its major components was performed on human glucocorticoids receptor (GR). Seventy-five components were identified in which longifolene (33.13%) and palmitic acid (9.34%) constituted the predominant components. No binding was observed on cannabinoid receptor type 1 (CB1), whereas mild binding was observed on cannabinoid receptor type 2 (CB2), *delta*, *kappa*, and *mu* receptors accounting for 2.9%, 6.9%, 10.9% and 22% binding. A significant in vivo activity was evidenced by reduction of the elevated malondialdehyde (MDA), nitric oxide (NO), myeloperoxidase (MPO), interleukin-6 (IL-6), and tumor necrosis factor-*α* (TNF-*α*) levels by 55.56%, 55.66%, 64.64%, 58.85% and 77.78% with concomitant elevation of superoxide dismutase (SOD) and catalase (CAT) activities comparable to BLM-treated group at 100 mg/kg body weight. In silico studies showed that palmitic acid exerted the fittest binding. PRO could serve as a potent anti-inflammatory natural candidate that should be supported by further clinical trials.

## 1. Introduction

Inflammation is the natural body response to injury of cells caused by numerous factors comprising infections, mechanical, and thermal injuries in addition to chemicals [1] that cause severe tissue damage and localized edema [2]. Meanwhile, essential oils represent volatile secondary metabolites that are normally produced within special cells or glands by medicinal plants. They are highly popular of possessing various biological activities comprising anti-inflammatory activity offering a relatively safe, effective, and low-cost medication [3]. On the contrary, the commonly used synthetic anti-inflammatory agent, non-steroidal anti-inflammatory drugs (NSAIDs), provoke a lot of serious side effects including gastro-intestinal ulceration, bleeding, renal dysfunction together with destruction of joint and cartilage owing to their nonselective prohibition of COX-1 and COX-2 enzymes [4].

*Pinus roxburghii* Sarg, belonging to family Pinaceae, is commonly named by “Chir pine” and is native to the Himalaya. It exists as a large ornamental tree nearly 30–50 m high possessing a trunk of about 2 m in diameter. It is found at 500 to 2500 m above the sea level growing gregariously [5]. Traditionally, it was widely employed as an antiseptic, diaphoretic, and a diuretic agent, in addition to exerting tonic and pronounced vermifuge and rubefacient activities [6,7]. The oil of the plant was greatly utilized in various herbal preparation for relieving inflammatory disorders [5].

Nowadays, it shows various prominent biological and pharmacological activities including antioxidant, anti-inflammatory, hepatoprotective, antidyslipidemic, in addition to alleviation of bronchitis, ear, eye, blood, throat, and skin diseases. Besides, its gum showed promising analgesic, aphrodisiac, anthelmintic, carminative, diuretic, expectorant, emmenagogue, and purgative activities [5,8,9].

The chief active constituents of the bark include *β*-sitostertol, ceryl alcohol, and friedelin, whereas abietic acid and isopimaric acid were obvious in xylem vessels. The predominant chemical constituents of *P. roxburghii* turpentine oil are *α*-pinene, *β*-pinene, car-3-ene, as well as longifolene to which many of its biological activities could be attributed [6]. Moreover, *Pinus roxburghii* needle oil from Pakistan showed nine components in which *α*-pinene, caryophyllene, and 3-carene predominate, while its stem oil formed of 17 components with the same three previously mentioned components but which differ in their relative quantities [10].

Although *Pinus* species are highly popular of possessing essential oils with an attractive medicinal value [11,12,13], few were found in the current literature concerning the chemical composition and the biological activity of the essential oil of *Pinus roxburghii* bark growing in Egypt. Thus, the foregoing study aimed to qualitatively and quantitatively identify the volatile oil composition isolated from *Pinus roxburghii* bark. Evaluation of its anti-inflammatory properties in vitro through assessing the inhibitory effect on the cannabinoids and opioids receptors was performed for the first time. Additionally, in vivo assessment using bleomycin-induced inflammation in *albino* mice was first to be adopted to consolidate its ethnopharmacological anti-inflammatory importance. Moreover, an in silico molecular docking study of the major essential oil components was performed on human glucocorticoids receptor (GR) in an effort to verify its potential mode of action.

## 2. Results and Discussion

### 2.1. Chemical Composition of P. roxburghii Bark Essential Oil (PRO)

A detailed investigation of the essential oil obtained from *P. roxburghii* bark cultivated in Egypt applying GC-FID and GC/MS techniques resulted in the identification of 75 components representing 89.65% of the total hydrodistilled oil. The oil is yellow in color with an aromatic odor, giving a yield of 0.022% *w*/*w* of dry plant. The chemical composition of the essential oil was listed in Table 1. The essential oil was predominated by sesquiterpene hydrocarbons accounting for 37.13% whereas monoterpenes, oxygenated monoterpenes, oxygenated sesquiterpenes, and other compounds represent 7.75%, 6.24%, 19.79% and 18.74% of the total oil components, respectively.

**Figure 1:**
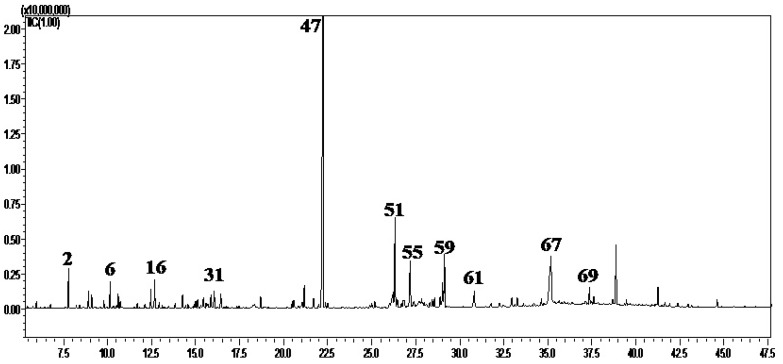
GC-chromatogram obtained with a Rtx-5MS column of the essential oil constituents isolated by hydrodistillation from *Pinus roxburghii* bark.

The major components were longifolene (33.13%), palmitic acid (9.34%), longicamphenylone (7.88%), 7(11)-Selinen-4*α*-ol (4.37%), and humulane-1,6-dien-3-ol (4.28%) as shown in Figure 1. Results verified that the sesquiterpene hydrocarbon, longifolene, is the prevalent component in *P. roxburghii* bark cultivated in different regions. However, the essential oil components vary quantitatively and qualitatively regarding the used organs as well as the geographical origin [10,14].

### 2.2. In Vitro Anti-Inflammatory Activity of PRO

Concerning the anti-inflammatory activity of the essential oil, it was previously reported that an effective anti-inflammatory therapy could be achieved by its concomitant administration with analgesics [15]. Analgesic agents achieved their targeted effects by acting with plethora of mechanisms on the central and/or peripheral nervous systems. These mechanisms may include the inhibition of prostaglandins synthesis through the interference with the cyclooxygenase and lypoxygenase pathways either peripherally or centrally. Moreover, prohibition of G-protein-mediated signal transduction, induction of endogenous opioid peptides, blockage of 5-hydroxytryptamine release, and inhibition of excitatory amino acids or *N*-methyl-d-aspartate receptor activation have been also reported [16,17]. Well-known analgesic drugs comprise paracetamol, NSAIDs exemplified by salicylates, in addition to cannabinoids and opioids, such as morphine. Synthetic anti-inflammatory agents, particularly NSAIDs, that are widely employed for the relief of pain and inflammation provoke a lot of serious side effects. Thus, in vitro analgesic and the anti-inflammatory activities herein were examined via acting on the cannabinoid receptors, CB1 that exists in the central nervous system and CB2 that is present in the peripheral tissues in addition to the opioid receptors [18]. Thus, the binding percentages of the essential oil on the cannabinoids (CB1 and CB2) and opioids (*mu*, *delta*, and *kappa*) receptors were evaluated. Results showed that no binding was observed on CB1 receptors, whereas mild to moderate binding was observed on CB2, *delta, kappa*, and *mu* receptors accounting for are 2.9%, 6.9%, 10.9%, and 22% binding respectively as recorded in Table S1.

### 2.3. In Vivo Anti-Inflammatory Activity of PRO

**Figure 2:**
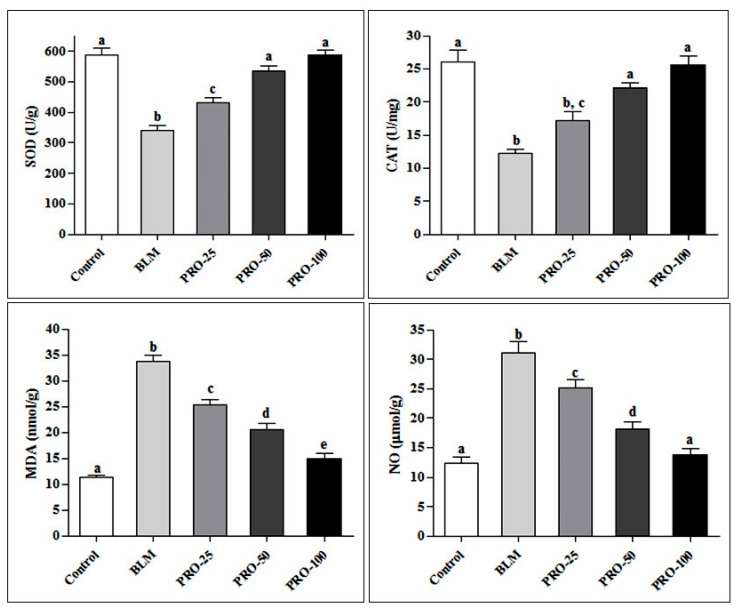
Effect of the PRO on oxidative stress markers (SOD, CAT, MDA, and NO) in lung tissue homogenate of BLM-treated mice. Different superscripts refer to significance at *p* ≤ 0.05 (*n* = 6).

Regarding in vivo anti-inflammatory effect, a potent effect of the essential oil in alleviating oxidative stress markers was observed. There was a significant increase in MDA and NO (nitric oxide) levels. Additionally, a pronounced decrease in SOD and CAT activities in the lung homogenate of BLM-treated group compared to the control group was observed. Administration of PRO significantly reduced the elevated MDA and NO levels by 55.56% and 55.66% compared to the BLM-treated group at a dose of 100 mg/kg body weight. Meanwhile, SOD and CAT were significantly increased in a dose-dependent manner with 72% and 108% increase relative to BLM-treated group at a dose of 100 mg/kg body weight, as recorded in Figure 2.

Additionally, the levels of proinflammatory cytokines MPO, IL-6, and TNF-*α* were significantly increased in the BLM-treated group compared to the control group. MPO increase was ameliorated by PRO at doses of 50 and 100 mg/kg, showing 36.49% and 64.64% decline comparable to the BLM-treated group in the previously mentioned doses, respectively. Moreover, the elevation of IL-6 and TNF-*α* levels were significantly lowered by PRO in a dose-dependent level, showing 58.85% and 77.78% decline relative to the negative control group at 100 mg/kg body weight, as shown in Table 2.

The histopathological assessments of pulmonary inflammation and fibrosis recorded in bleomycin and other treated groups are demonstrated in Table 3.

**Figure 3:**
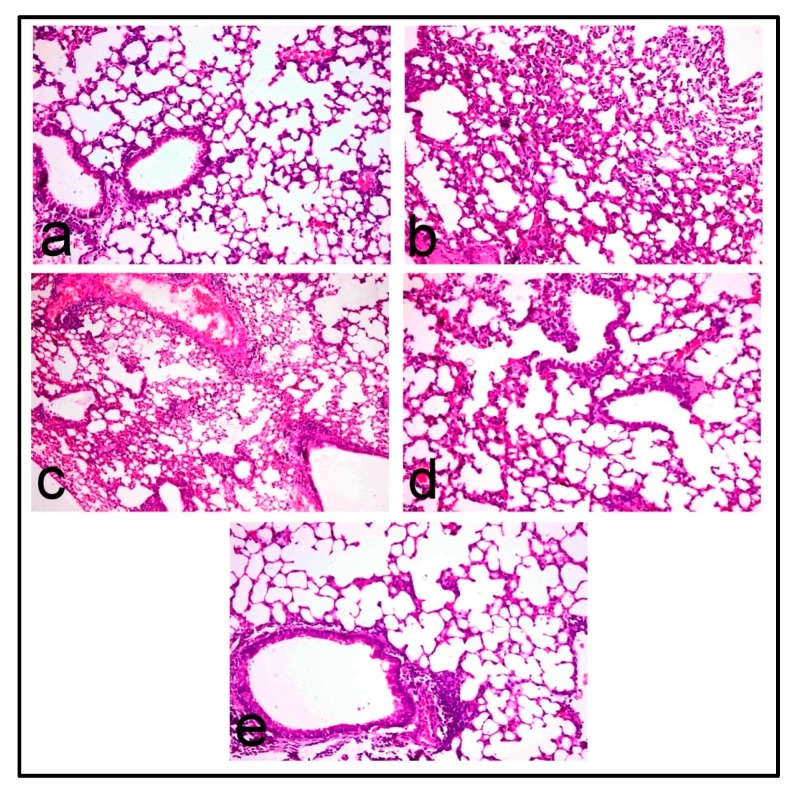
Histopathological images of BLM-induced lung fibrosis in mice; Lung of (**a**) control mice showing normal pulmonary parenchyma; (**b**) BLM-treated mice showing marked thickening of alveolar wall and interstitial tissue by proliferating mononuclear cells mostly lymphocytes and macrophages and fibroblastic proliferation; (**c**) PRO-25 treated mice showing interstitial inflammatory cell infiltrates; (**d**) PRO-50 treated mice showing less distorted pulmonary parenchyma with mild interstitial inflammatory cell infiltration, and (**e**) PRO-100 treated mice showing normal pulmonary parenchyma. (H&E, 200×).

**Figure 4:**
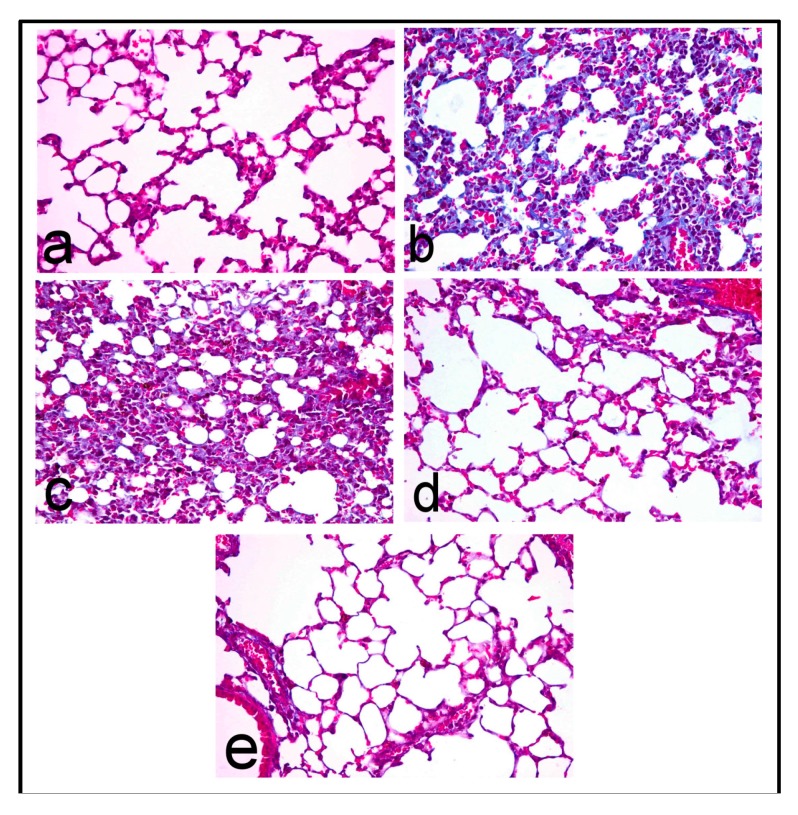
Lung of (**a**) control mice showing no collagen deposition; (**b**) BLM-treated mice showing intensely blue collagen fibers; (**c**) PRO-25 treated mice showing blue stained collagen fibers; (**d**) PRO-50 treated mice showing no collagen deposition; and (**e**) PRO-100 treated mice showing no collagen deposition. (Masson’s trichrome stain, 400×).

Lungs of control mice revealed normal pulmonary parenchyma (Figure 3a) with no evidence of interstitial fibrosis which is confirmed by Masson’s trichrome stain (Figure 4a). In contrast, lungs of BLM-treated mice revealed distortion of pulmonary parenchyma with alveolar and interstitial fibrosis scored 2.60 ± 0.24, which is characterized by marked thickening of alveolar walls and interstitial tissue by proliferating mononuclear cells, mostly lymphocytes and macrophages, as well as fibroblastic proliferation (Figure 3b) with collagen deposition that appeared intensely blue in Masson’s trichrome stained sections (Figure 4b). Most alveoli appeared partially collapsed with partial obliteration of alveolar lumina.

Mild improvement of pulmonary inflammation and fibrosis was demonstrated in the PRO-25 pretreated group, which scored 2.20 ± 0.37, in which the interstitial inflammatory infiltrates (Figure 3c) and collagen deposition, which appeared blue in Masson’s trichrome stained sections (Figure 4c), were evident. Marked regression of fibrosis and interstitial inflammatory infiltrates was recorded in the PRO-50 and PRO-100 pretreated groups with lesion scores 1.40 ± 0.24 and 1.20 ± 0.20, respectively. The pulmonary parenchyma of the PRO-50 pretreated group was less distorted with mild interstitial inflammatory cell infiltration (Figure 3d) and no collagen deposition, which is confirmed in Masson’s trichrome stained sections (Figure 4d). Lung of PRO-100 pretreated groups appeared normal (Figure 3e) and greatly similar to the control group, and the interstitial inflammatory infiltrates were confined to focal areas which affect less than 25% of the lung parenchyma. Masson’s trichrome stain confirmed no collagen deposition in nearly all of the examined sections (Figure 4e).

### 2.4. In Silico Molecular Modelling Study of PRO Major Constituents in GR

Glucocorticoids have long been used in the alleviation of patients suffering from pulmonary fibrosis via prohibition of procollagen synthesis, both in connective tissues and in fibroblasts, in addition to reduction of pulmonary parenchymal cell proliferation. This was explained in virtue of the reversal of type I procollagen mRNA partitioning in polysomes together with reversing stimulation of procollagen synthesis induced by bleomycin [19,20]. Thus, molecular docking of the major essential oil components was done within the active site of human glucocorticoids receptors and showed that most of them have affinity to the binding site as compared to the potent anti-inflammatory GR agonist, dexamethasone, and thus can exert a potent anti-inflammatory activity. The free binding energy of these compounds was chosen as a comparison factor in the docking experiments, as recorded in Table 4.

**Figure 5:**
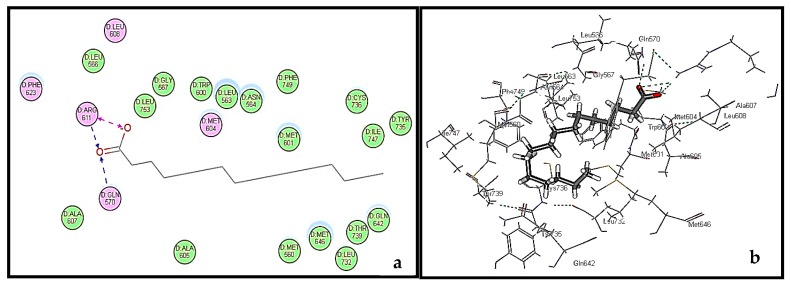
2D binding mode (**a**) and 3D binding mode (**b**) of palmitic acid in the active site of human glucocorticoid receptors (GR).

The validity of the docking study was confirmed by comparing and alignment of the best docked poses of the lead compound, dexamethasone, with the lead conformer co-crystallized with GR. Results revealed a good alignment between them (RMSD = 0.60 A°), confirming the validity of the experiment (Figure S1). Among all the docked major constituents, palmitic acid exerted the fittest binding to GR as evidenced from its free binding energy that approaches that of dexamethasone, being −60.28 and 61.08 kcal/mol, respectively. Examining the active site of GR, it showed the presence of many hydrophobic residues. Binding of palmitic acid in the active pocket of human glucocorticoid receptors (GR) is illustrated in Figure 5b. Its firm binding could be explained by virtue of the formation of three hydrogen bonds with the amino acid residues of Arg611 and Gly570, in addition to the hydrophobic interaction with the GR as shown in Figure 5a,b. This may highlight that the essential oil of *Pinus roxburghii* could partly act in a manner similar to glucocorticoids, owing to the agonist effect of its major constituents within the active site, similarly to dexamethasone. Glucocorticoids are highly potent in relieving inflammation owing to their pleiotropic effects observed on the glucocorticoid receptor affecting multiple signaling pathways. Unfortunately, this pleiotropic behavior may widely affect many metabolic processes causing growth retardation in children and affecting immune system. In addition, hypertension, suppression of wound healing, and osteoporosis are among the common adverse effects of prolonged glucocorticoid regimen [21]. However, essential oil of *Pinus roxburghii* could alleviate inflammation with minimal observed adverse effects.

## 3. Materials and Methods

### 3.1. Plant Material

*Pinus roxburghii* Sarg. Bark (Pinaceae) was collected from El-Orman Botanical Garden (Giza, Egypt) on April 2014 and authenticated by Mrs. Terease Labib, Consultant of Plant Taxonomy at Ministry of Agriculture, El-Orman Botanical Garden and National Gene Bank, Giza, Egypt. A voucher specimen was deposited in the herbarium of the Pharmacognosy Department, Faculty of Pharmacy, Ain Shams University (P-PR-7).

### 3.2. Essential Oil Preparation

Volatile oil of *Pinus roxburghii* bark was obtained by hydrodistillation (250 g) for 4 h using a Clevenger-type apparatus. The oil was dried over anhydrous sodium sulfate and kept in a sealed vial at −30 °C for further analyses. The yield was performed in triplicate, determined based on the initial dried plant weight, and expressed in % (*w*/*w*).

### 3.3. Gas Chromatography

#### 3.3.1. GC/FID Analysis

GC/FID analysis was performed using a Shimadzu GC-17A gas chromatograph (Shimadzu Corporation, Kyoto, Japan) supplied with FID detector and a DB-5 fused-bonded cap column (Phenomenex; 29 m × 0.25 mm i.d., film thickness 0.25 µm; Torrance, CA, USA). The oven temperature was programmed isothermal at 50 °C for 2 min, then raised from 50 to 300 °C at 7 °C/min, and finally held isothermal at 300 °C for 10 min; injector temp., 250 °C; detector temp., 300 °C; carrier gas, He (1.5 mL/min); with split mode (split ratio, 1:20). The sample 0.1 μL was injected automatically to the chromatograph using AOC-20i auto sampler. Class GC 10^®^software (Shimadzu Corporation, Kyoto, Japan) was used for recording and integrating the chromatograms. Average areas under the peaks of three independent chromatographic runs were used for calculating the % composition of each component (total peak area = 100%).

#### 3.3.2. GC/MS Analysis

Mass spectrum was recorded using Shimadzu GC-2010 plus gas chromatograph (Shimadzu Corporation, Kyoto, Japan) coupled to a quadrupole mass spectrometer Shimadzu QP-2010 equipped with Rtx-5MS fused bonded column (30 m × 0.25 mm i.d. × 0.25 µm film thickness) (Restek, Bellefonte, PA, USA) equipped with a split-splitless injector. The capillary column was directly coupled to a quadrupole mass spectrometer. The initial column temperature was kept at 45 °C for 2 min (isothermal) and programmed to 300 °C at a rate of 5 °C/min, and kept constant at 300 °C for 5 min (isothermal). Detector and injector temperatures were 300 and 250 °C, respectively. Helium carrier gas flow rate was 2 mL/min. Mass spectra were recorded applying the following condition: (equipment current) filament emission current, 60 mA; ionization voltage, 70 eV; ion source, 200 °C. Diluted samples (0.5% *v*/*v*) were injected with split mode (split ratio 1:15). The sample (1 μL) was injected automatically to the chromatograph using AOC-20i auto sampler. GC solution^®^ software ver. 2.4 (Shimadzu Corporation, Kyoto, Japan) was used for recording and integrating the chromatograms. Volatile components were identified by comparing their retention indices (RI) and mass spectra with those built in libraries (NIST Mass Spectral Library (December 2005), Wiley Registry of Mass Spectral Data 8th edition) and literature [22,23].

### 3.4. In Vitro Anti-Inflammatory Activity Evaluation

#### 3.4.1. Cannabinoid Receptor Binding Assay

For each assay, non-specific binding was determined using 10 μM of CP-55,940 as a positive control. Total, specific, and non-specific binding were used to calculate the binding percentage of the non-labeled control to CB1 and CB2 receptors. The membrane concentration exhibiting good binding percentage (>90%) and total binding with high signal (thousands of CPM) was used as the optimal membrane concentration for the assay [24].

#### 3.4.2. Opioid Receptor Binding Assay

Human embryonic kidney cells (HEK293) stably transfected with human opioid receptor subtypes μ, δ, or κ were used to perform the opioid receptor binding assays. For each assay, non-specific binding was determined using 10μM of a positive control [U-69,593 [phenyl-3,4-3H] (κ), Enkephalin DPDPE [Tyrosyl-3,5-3H(N)] (δ), or DAMGO [Tyrosyl-3,5-3H(N)] (µ). The dissociation constant [4] for ligands for each receptor was established through a membrane evaluation and saturation binding experiment. Total, specific, and non-specific binding were used to calculate the binding percentage of the non-labeled control to receptor. The membrane concentration exhibiting good binding percentage (>90%) and total binding with high signal (thousands of CPM) was used as the optimal membrane concentration for the assay [24].

Data of the two assays were analyzed by a non-linear curve fit model using GraphPad Prism 5.04 software (GraphPad, La Jolla, CA, USA) and the Kd value was calculated. General screening was performed using the optimal concentration of membrane with a radioligand concentration less than or equals to the Kd. Each compound was tested at 10 μM in triplicates. The assays were performed as stated above. Non-specific binding was subtracted from total binding to find specific binding. Percent binding was determined using the following equation:

$$\%\;\mathrm{binding}=100-\left(\frac{\mathrm{Compound}\;\mathrm{CPM}-\mathrm{nonspecific}\;\mathrm{CPM}}{\mathrm{specific}\;\mathrm{CPM}}\right)\times 100$$

The competitive binding assay was performed using the optimal concentration of membrane with radioligand concentration equal to the Kd, and 12 concentrations of each compound ranging from 0.032 to 10,000 μM. Each compound was tested in triplicate. The assays were performed as stated above. The IC_50_ and Ki values were calculated by a non-linear curve fit model using GraphPad Prism 5.0 software.

### 3.5. In Vivo Anti-Inflammatory Activity Evaluation

#### 3.5.1. Drugs, Chemicals, and Kits

Bleomycin in the form of Blecocip, vial, 15 units was obtained from (Cipla Ltd., Mumbai, India). Kits for evaluating the lipid peroxidation marker, malondialdehyde (MDA), as well as those for antioxidant parameters represented by superoxide dismutase (SOD) and catalase (CAT) were bought from Biodiagnostics Co. (Cairo, Egypt). The pro-inflammatory cytokines (IL-6, TNF-*α*) were purchased from R&D Systems GmbH (Wiesbaden, Germany). Myeloperoxidase (MPO) ELISA kits was brought from Hycult Biotechnology, Plymouth Meeting, PA, USA. All other chemicals used were of analytical grade.

#### 3.5.2. Animals

Thirty male Swiss albino mice, ten-weeks-old, weighing about 25 ± 2 g, were purchased from the Egyptian Organization for Biological Products and Vaccines, Giza, Egypt. The animals were reared at Experimental Animal Unit at Faculty of Veterinary Medicine, Suez Canal University, Ismailia, Egypt. They were kept on a 12-h light/dark cycle in a maintained temperature (24 ± 2 °C) and humidity (50 ± 5%) conditions. All procedures in this animal study were performed according the guidelines of NIH and approved by Animal Research Ethics committee of Faculty of Veterinary Medicine, Suez Canal University, Ismailia, Egypt (Approval Number:201613).

#### 3.5.3. Experimental Design

The animals were divided into 5 groups, each of 6 mice. The first group was given saline and kept as control. The mice in second group were intraperitoneally injected with bleomycin (BLM) at a dose of 15 mg/kg twice a week for four consecutive weeks [25]. The third, fourth, and fifth groups were intraperitoneally injected with essential oil (PRO) at doses of 25, 50, 100 mg/kg body weight, respectively. Five days after the last BLM dose, the mice were sacrificed, and lung tissues were harvested. The right lung was rapidly excised, weighed, frozen, and kept at −80 °C until used for the assessment of lipid peroxidation marker, MDA, antioxidant enzyme activities; SOD and CAT, as well as the assay of the proinflammatory cytokines; interleukin-6 (IL-6) and tumor necrosis factor alpha (TNF-*α*). Moreover, myeloperoxidase (MPO) was estimated as indicator for leucocytic adhesion and infiltration.

#### 3.5.4. Evaluation of Lipid Peroxidation and Oxidative Stress Markers

Lung lobes were thawed at 4 °C, homogenized on ice in 50 mM Tris-HCl buffer containing 180 mM KCl and 10 mM EDTA, final pH 7.4, and then centrifuged at 10,000 rpm, 4 °C, for 30 min. Lung homogenates were centrifuged at 860 rpm for 20 min at 4 °C and the resultant supernatant was frozen at −20 °C and used for assays of different oxidative stress markers. Superoxide dismutase (SOD) was determined according to Nishikimi et al., 1972 [26], catalase (CAT) according to Aebi, 1974 [27], malondialdehyde (MDA) according to Mihara and Uchiyama, 1978 [28].

#### 3.5.5. Evaluation of the Inflammatory Markers 

Lung homogenate supernatants were used for assay of IL-6 and TNF-*α* using Mouse IL-6 and TNF-*α* ELISA kit following the protocol provided by the manufacturer. Values are expressed as pg/mL. Myeloperoxidase (MPO) activity was evaluated as an indicator for leukocyte infiltration and adhesion in many tissues, including the lung. Estimation of MPO activity was carried out according to the manufacturer protocol, and its values are expressed as pg/g tissue.

#### 3.5.6. Histological Study

Ten lung sections per group were fixed in 10% neutral formalin, washed, and embedded in paraffin blocks. 5 µm thick sections were stained with H&E to demonstrate the pulmonary fibrosis and inflammation, and Masson’s trichrome stains to demonstrate collagen deposition. Semi-quantitative lesion scoring was performed, according to the method described by Okazaki et al., 2001 [29], to evaluate the pulmonary inflammation and fibrosis in which: 0, normal lung tissue; 1, pulmonary inflammation and fibrosis affecting <25% of the lung; 2, pulmonary lesions affecting 25–50% of the lung; and 3, lesions affecting >50% of the lung. The mean of the lesion scores was estimated for five lung sections per mouse. 

#### 3.5.7. Statistical Analysis

Data for the in vivo experiment are presented as mean ± S.E. Statistical significance of the data was analyzed using SPSS program (Statistical Package for Social Science) version 16. For comparison, One-Way analysis of variance (ANOVA) test and post-comparison was carried out with Duncan’s Multiple Range Test for post hoc analysis. Statistical significance was acceptable to a level of *p* ≤ 0.05. 

### 3.6. Molecular Modelling Study

The X-ray crystal structure of human glucocorticoid receptor (PDB ID1M2Z; 2.5 A°) co-crystalized with its ligand, dexamethasone, was downloaded from protein data bank (www.pdb.org). The structure of the enzyme was established using the default protein preparation protocol of Accelry’s discovery studio 2.5 (Accelrys^®^, Inc., San Diego, CA, USA). Molecular modelling studies were done using Accelry’s discovery studio 2.5 (Accelrys^®^, Inc., San Diego, CA, USA) according to what was previously reported [30,31,32] and the binding free energies were calculated applying the following equation:

ΔG_binding_ = E_complex_− (E_GR_ + E_ligand_)

where
ΔG _binding_The ligand–enzyme interaction binding energyE _complex_The potential energy for the complex of GR bound with the ligandE _GR_The potential energy of the protein aloneE _ligand_The potential energy for the ligand alone


## 4. Conclusions

In conclusion, the investigations of the current study revealed that the essential oil of *P. roxburghii* bark could serve as a potent anti-inflammatory natural candidate as evidenced from the in vivo study. Molecular docking of the major oil components on human glucocorticoid receptors showed affinity to the binding site as compared to the dexamethasone, the highly potent anti-inflammatory agent. This further consolidates its ethnomedicinal value as an anti-inflammatory agent. Its probable mode of action could be partly interpreted by virtue of its agonistic effect on the glucocorticoid receptor and hence could be used as a promising natural anti-inflammatory candidate. Nevertheless, these findings should be supported by further in vitro, in vivo, and finally clinical trials to confirm the claimed activity.

## Figures and Tables

**Table 1:** Volatile constituents identified in *Pinus roxburghii* bark essential oil (PRO).

Component		RI		Content (%)	Identification Method
		Calculated	Reported		
**1**	*α*-Thujene	922	922	tr.	MS, RI
**2**	*α*-Pinene	929	929	2.21	MS, RI
**3**	Camphene	945	945	0.17	MS, RI
**4**	1-Isopropyl-4-methylenebicyclo[3.1.0]hex-2-ene	951	952	0.19	MS, RI
**5**	1,2,4-Trimethylbenzene	995	994	tr.	MS, RI
**6**	3-Carene	1013	1013	1.5	MS, RI
**7**	Isocineole	1018	1018	tr.	MS, RI
**8**	*α*-Terpinene	1019	1019	0.09	MS, RI
**9**	*ρ*-Cymene	1025	1025	0.25	MS, RI
**10**	*o*-Cymene	1027	1026	0.87	MS, RI
**11**	d-Limonene	1031	1031	0.37	MS, RI
**12**	Eucalyptol (*p*-Cineole)	1034	1034	tr.	MS, RI
**13**	*trans*-Bicyclo[4.4.0]Decane	1057	1056	0.1	MS, RI
**14**	*τ*-Terpinene	1062	1062	0.32	MS, RI
**15**	2-Methyldecane	1066	1063	tr.	MS, RI
**16**	Terpinolene	1094	1093	1.97	MS, RI
**17**	*n*-Undecane	1102	1100	0.34	MS, RI
**18**	*n*-Nonanal	1108	1108	0.17	MS, RI
**19**	*α*-Thujone	1110	1110	0.06	MS, RI
**20**	Fenchol, exo-	1119	1119	0.14	MS, RI
**21**	l*-trans*-Pinocarveol	1144	1143	0.95	MS, RI
**22**	Camphor	1150	1150	0.18	MS, RI
**23**	Isoborneol	1163	1162	tr.	MS, RI
**24**	*α*-Phellandren-8-ol	1166	1166	0.23	MS, RI
**25**	Pincarvone	1169	1164	0.46	MS, RI
**26**	Borneol	1172	1173	tr.	MS, RI
**27**	*p*-Acetyltoluene	1178	1179	0.22	MS, RI
**28**	1-Terpinen-4-ol	1183	1183	0.84	MS, RI
**29**	*p*-Cymen-8-ol	1187	1187	0.31	MS, RI
**30**	*τ*-Terpineol	1196	1192	0.95	MS, RI
**31**	Myrtenal	1202	1204	1.4	MS, RI
**32**	*n*-Decanal	1209	1209	tr.	MS, RI
**33**	*cis*-Carveol	1226	1226	0.12	MS, RI
**34**	Acetophenone, 2′,4′-dimethyl	1229	1230	0.09	MS, RI
**35**	Cumin aldehyde	1248	1248	0.16	MS, RI
**36**	*n-*Nonanoic acid	1282	1282	tr.	MS, RI
**37**	Thymol	1304	1304	tr.	MS, RI
**38**	Carvacrol	1311	1311	tr.	MS, RI
**39**	2,4-Decadienal	1324	1322	tr.	MS, RI
**40**	*α*-Terpinyl acetate	1357	1356	0.44	MS, RI
**41**	*α*-Longipinene	1360	1360	0.63	MS, RI
**42**	*n*-Decanoic acid	1376	1376	tr.	MS, RI
**43**	Longicyclene	1381	1380	2.18	MS, RI
**44**	*α*-Ylangene	1385	1385	tr.	MS, RI
**45**	(+)-Sativen	1399	1391	0.87	MS, RI
**46**	*β*-Caryophyllene	1411	1411	0.32	MS, RI
**47**	d-longifolene	1419	1418	33.13	MS, RI
**48**	*n*-Undecanoic acid	1444	1445	tr.	MS, RI
**49**	*α*-Humulene	1467	1468	tr.	MS, RI
**50**	Dodecanoic acid	1576	1578	2.53	MS, RI
**51**	(+)-Longicamphenylone	1580	1563	7.88	MS, RI
**52**	Globulol	1586	1587	0.72	MS, RI
**53**	Caryophyllene oxide	1594	1594	1.66	MS, RI
**54**	Viridiflorol	1608	1609	tr.	MS, RI
**55**	Humulane-1,6-dien-3-ol	1615	1619	4.28	MS, RI
**56**	Tridecanoic acid	1643	1645	0.42	MS, RI
**57**	Longiverbenone	1670	1678	tr.	MS, RI
**58**	*α*-Santalol	1689	1683	0.88	MS, RI
**59**	7(11)-Selinen-4*α*-ol	1700	1688	4.37	MS, RI
**60**	Pentadecanal	1722	1717	tr.	MS, RI
**61**	Myristic acid	1774	1774	1.84	MS, RI
**62**	Octadecane	1802	1800	0.06	MS, RI
**63**	*n*-Pentadecanoic acid	1873	1872	0.97	MS, RI
**64**	Nonadecane	1904	1900	0.16	MS, RI
**65**	Palmitic acid, methyl ester	1926	1926	tr.	MS, RI
**66**	9-Hexadecenoic acid	1953	1957	0.51	MS, RI
**67**	Palmitic acid	1979	1975	9.34	MS, RI
**68**	*n*-Eicosane	2002	2000	tr.	MS, RI
**69**	1-Octadecanol	2096	2086	1.23	MS, RI
**70**	*n-*Heneicosane	2109	2100	0.57	MS, RI
**71**	*n*-Docosane	2211	2200	tr.	MS, RI
**72**	*n*-Tricosane	2304	2300	tr.	MS, RI
**73**	*n*-Tetracosane	2403	2400	tr.	MS, RI
**74**	*n*-Pentacosane	2509	2500	tr.	MS, RI
**75**	*n*-Hexacosane	2610	2600	tr.	MS, RI
Monoterpene hydrocarbons				7.75	
Oxygen containing monoterpene				6.24	
Sesquiterpene hydrocarbons				37.13	
Oxygen containing sesquiterpene				19.79	
Others				18.74	
Total identified components				89.65	

**Table 2:** Effect of the PRO on the inflammatory markers in lung tissue homogenate of BLM-treated mice.

	Control	BLM	PRO-25	PRO-50	PRO-100
**MPO (pg/g)**	23.21 ± 1.07 ^a^	88.99 ±3.30 ^b^	78.87 ± 5.47 ^b^	56.52 ± 2.31 ^c^	31.47 ± 2.36 ^a^
**IL-6(pg/g)**	192.63 ± 14.85 ^a^	775.38 ± 44.07 ^b^	640.46 ± 16.94 ^c^	453.46 ± 29.04 ^d^	319.05 ± 25.06 ^e^
**TNF-*α* (pg/mL)**	47.49 ± 2.84 ^a^	377.53 ± 16.57 ^b^	235.51 ± 14.71 ^c^	169.76 ± 9.59 ^d^	83.87 ± 4.43 ^e^

Different superscripts within the same row refer to significance at *p* ≤0.05 (*n* = 6).

**Table 3:** Effect of the PRO on pathological score of pulmonary inflammation and fibrosis in BLM-treated mice.

Group	Score
Saline	0.20 ± 0.20 ^a^
BLM	2.60 ± 0.24 ^c^
PRO-25	2.20 ± 0.37 ^c^
PRO-50	1.40 ± 0.24 ^b^
PRO-100	1.2 0 ± 0.20 ^b^

Results expressed as mean ± SD. Different superscripts within the same column were significantly different.

**Table 4:** Binding of the major PRO components to human glucocorticoid receptor (GR) using molecular modeling experiments as manifested by binding energy values (ΔG _binding_) expressed in kcal/mol.

Compound Name	ΔG _binding_
Dexamethasone	−61.0836
Palmitic acid	−60.2838
1-Octadecanol	−55.3628
Myristic acid	−54.4668
Dodecanoic acid	−49.2119
7(11)-Selinen-4-*α*-ol	−35.5702
Caryophyllene oxide	−34.8706
Humulane-1,6-dien-3-ol	−32.9638
Longicyclene	−31.6995
d-longifolene	−30.3795
Longicamphenylone	−30.0035
Myrtenal	−26.4264
*α*-Pinene	−21.7649
Terpinolene	−21.3438
3-Carene	−21.2806

## Supplementary Materials

Supplementary materials are available online.
