# Supplementary material for: Chemical Composition of Pinus roxburghii Bark Volatile Oil and Validation of Its Anti-Inflammatory Activity Using Molecular Modelling and Bleomycin-Induced Inflammation in Albino Mice
Source: Molecules. 2017 Aug 29;22(9):1384. doi: 10.3390/molecules22091384 (PMC6151475; doi:10.3390/molecules22091384)
Supplement: Supplementary file 1 [file molecules-22-01384-s001.pdf]

Supplementary data

Chemical Composition of *Pinus roxburghii* Bark Volatile Oil and Validation of Its Anti-Inflammatory Activity Using Molecular Modelling and Bleomycin-Induced Inflammation in *Albino* Mice

Rola M. Labib [1,2,*], Fadia S. Youssef [1], Mohamed L. Ashour [1], Mohamed M. Abdel-Daim [3] and Samir A. Ross [2,4]

[1] Department of Pharmacognosy, Faculty of Pharmacy, Ain-Shams University, Cairo 11566, Egypt; fadiayoussef@pharma.asu.edu.eg (F.S.Y.); ashour@pharma.asu.edu.eg (M.L.A.)

[2] National Center for Natural Products Research, University of Mississippi, University, MS 38677, USA; sross@olemiss.edu

[3] Department of Pharmacology, Faculty of Veterinary Medicine, Suez Canal University, Ismailia 41522, Egypt; abdeldaim.m@vet.suez.edu.eg

[4] Department of BioMolecular Sciences, School of Pharmacy, University of Mississippi, University, MS 38677, USA

* Correspondence: rolamilad@pharma.asu.edu.eg; Tel.: +20-1-001971922

Figure S1: (**a**) Alignment of the X-ray bioactive conformer with the best fitted pose of the lead compound (dexamethasone); (**b**) binding of palmitic acid within the active pocket of human glucocorticoid receptors (GR).
Table S1: The binding percentage of PRO on the cannabinoids and opioids receptors.

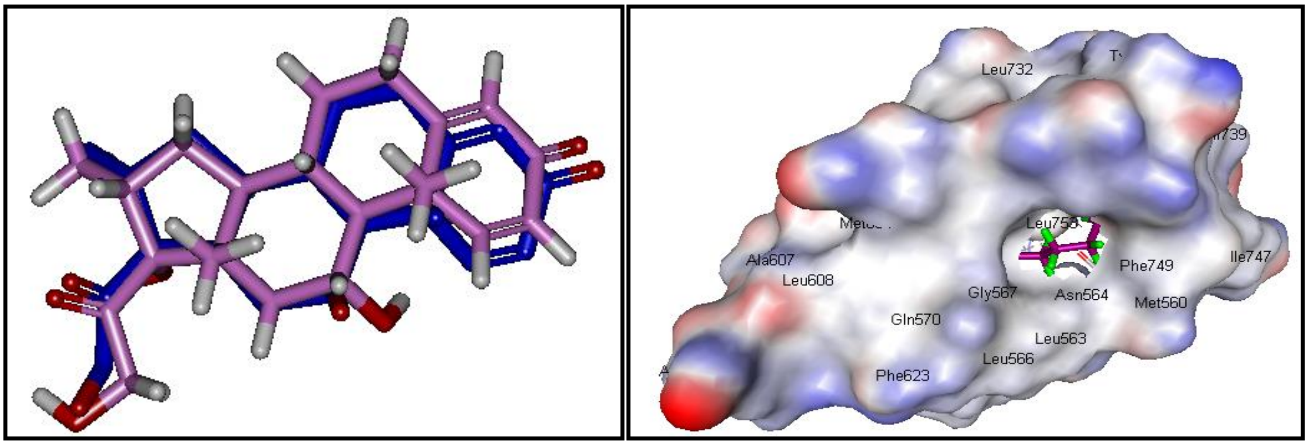

Figure S1: (**a**) Alignment of the X-ray bioactive conformer with the best fitted pose of the lead compound (dexamethasone); (**b**) binding of palmitic acid within the active pocket of human glucocorticoid receptors (GR).

Table S1: The binding percentage of PRO on the cannabinoids and opioids receptors.

Receptor	CB1	CB2	*Delta*	*Kappa*	*Mu*
**PRO**	**NA**	2.9	6.9	10.9	22.0

NA: no activity
